# Supplementary material for: The Attenuation of Insulin/IGF-1 Signaling Pathway Plays a Crucial Role in the Myo-Inositol-Alleviated Aging in Caenorhabditis elegans
Source: Int J Mol Sci. 2023 Mar 24;24(7):6194. doi: 10.3390/ijms24076194 (PMC10094297; doi:10.3390/ijms24076194)
Supplement: Supplementary file 1 [file ijms-24-06194-s001.zip › ijms-2256222-supplementary.pdf]

Supplementary materials

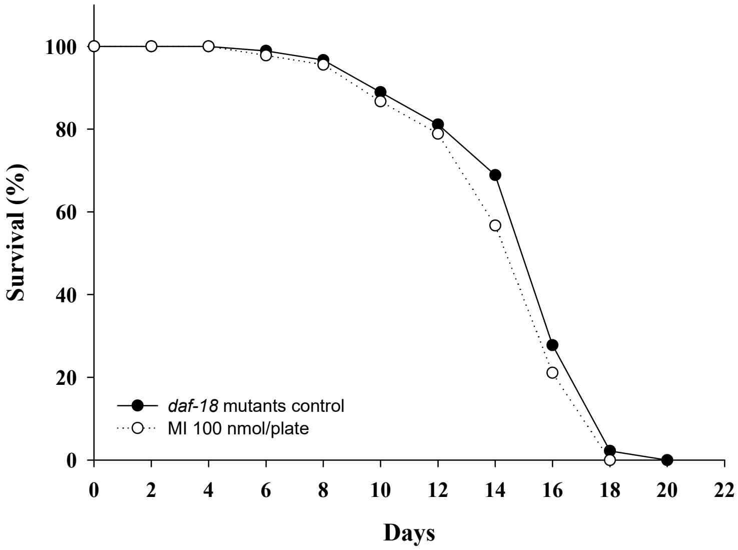

Figure S1. Effects of the myo-inositol (MI) on the lifespan of the DAF-18 mutants. The mutants (90 worms per group) were grown on plates containing with or without 100 nmol/plate of MI. The surviving worms were counted every two days. The surviving percentages were obtained and the results were analyzed by the log-rank test with the SPSS software. The results are from a representative experiment out of two independent experiments.

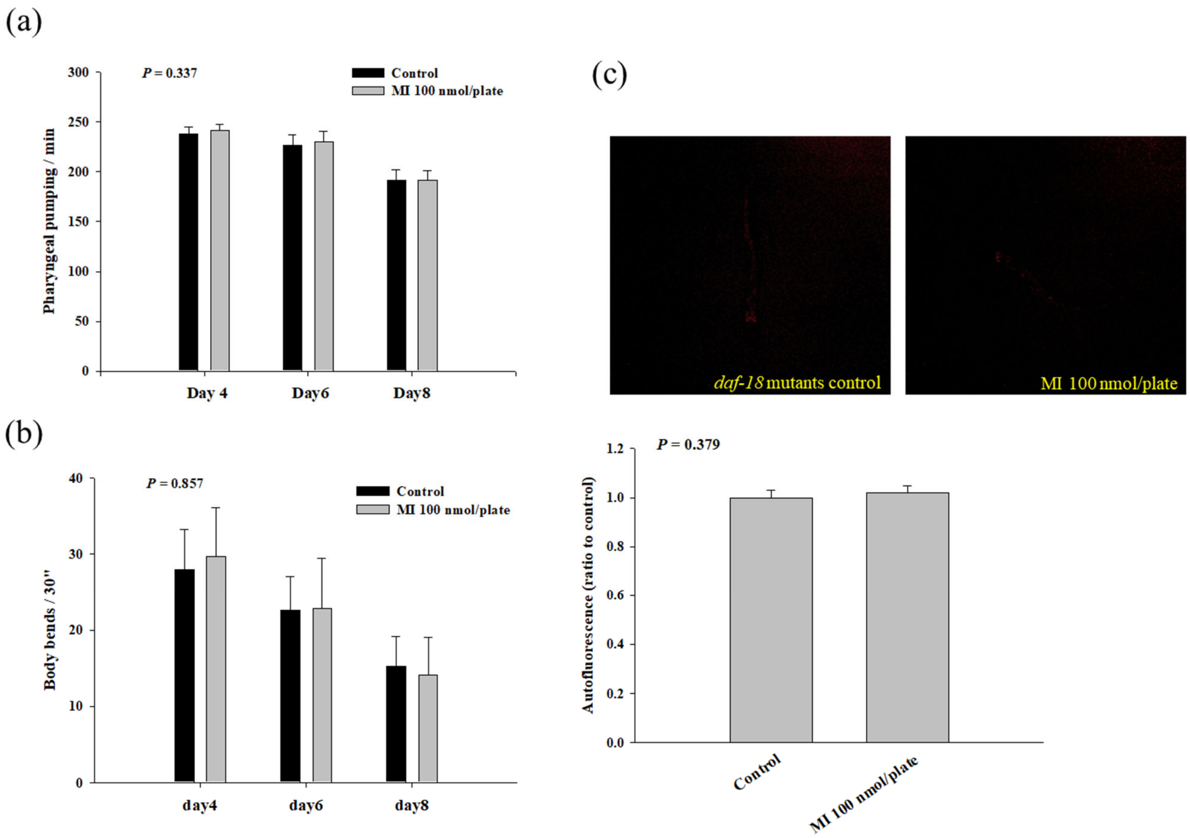

Figure S2. Effects of the myo-inositol (MI) on the pharyngeal pumping, body bends, and autofluorescence of the DAF-18 mutants. The mutants (90 worms per group) were grown on plates containing with or without 100 nmol/plate of MI. On the fourth, sixth and eighth days, and the 10th day of life, (**a**) the pharyngeal pumping (n = 10), (**b**) body bends (n = 10) and (**c**) autofluorescence (upper panel: photos; lower panel: quantitative results, n = 3) were determined as described in Methods. Values are presented as mean ± SD, and *p* values are shown in the figures. The results are from a representative experiment out of two independent experiments.

(**a**)

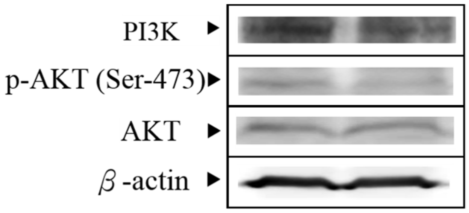

(**b**)

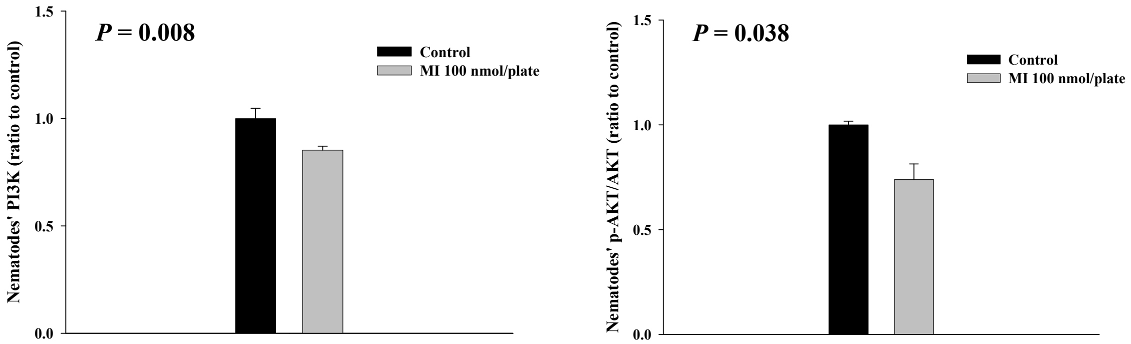

Figure S3. Effects of the myo-inositol (MI) on the expressions of nematodes' PI3K, and the phosphorylation of nematodes' AKT. After being treated with 100 nmol/plate of MI for five days, the nematodes were obtained. 10mg of nematodes in wet weight was used to prepare worm lysate. The protein expressions or protein phosphorylation level in the worm lysate were detected by the Western blot method as described in Methods. (**a**) a representative result of the Western blotting; (**b**) the quantitative results of the Western blotting (n = 3). Values are presented as mean ± SD, and *p* values between the two groups are shown.
